# Supplementary material for: Evaluation of a Triage Checklist for Mild COVID-19 Outpatients in Predicting Subsequent Emergency Department Visits and Hospitalization during the Isolation Period: A Single-Center Retrospective Study
Source: J Clin Med. 2022 Sep 16;11(18):5444. doi: 10.3390/jcm11185444 (PMC9506197; doi:10.3390/jcm11185444)
Supplement: Supplementary file 1 [file jcm-11-05444-s001.zip › jcm-1895254-supplementary.pptx]

## Slide 1
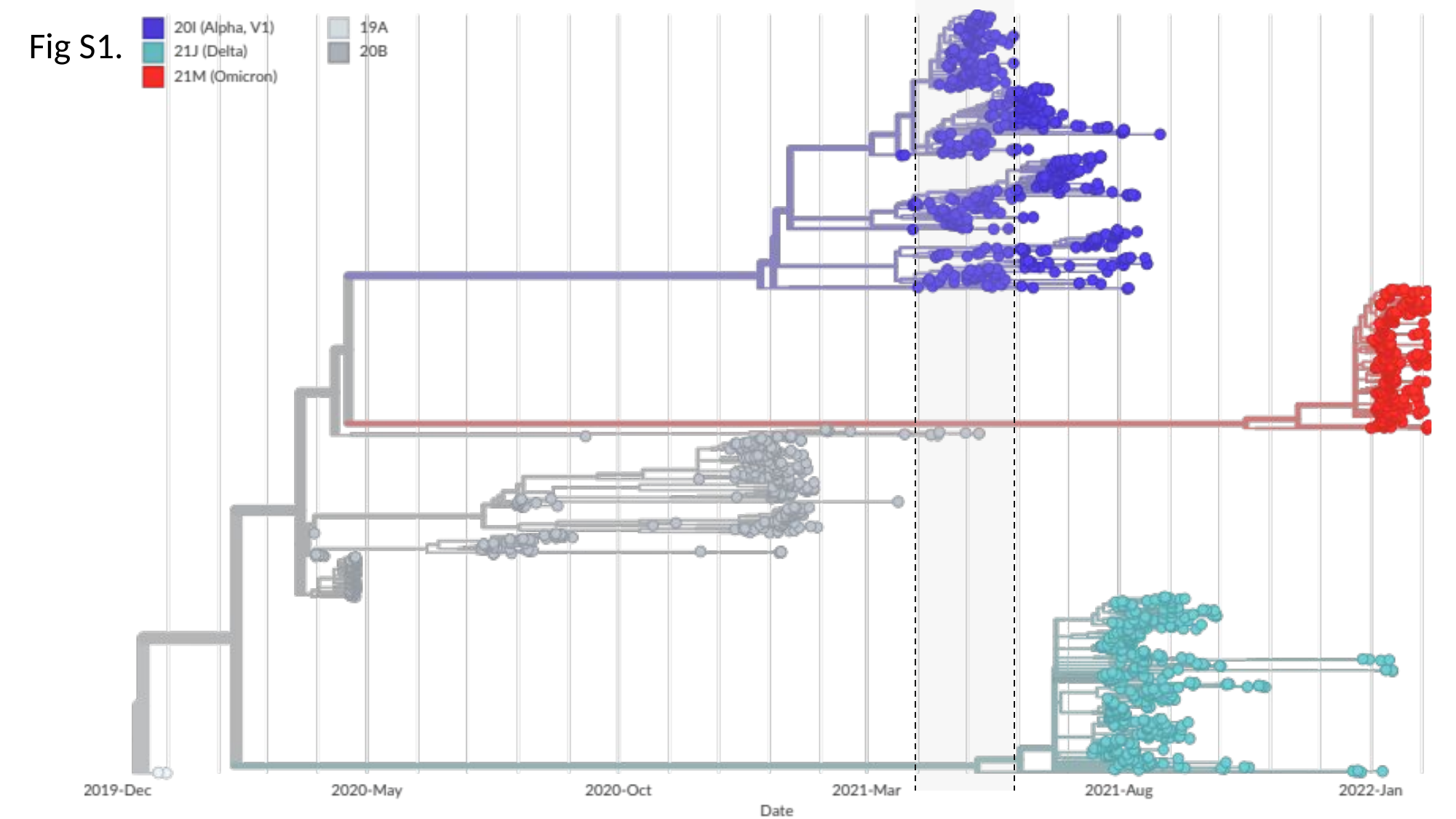

Fig S1.

## Slide 2
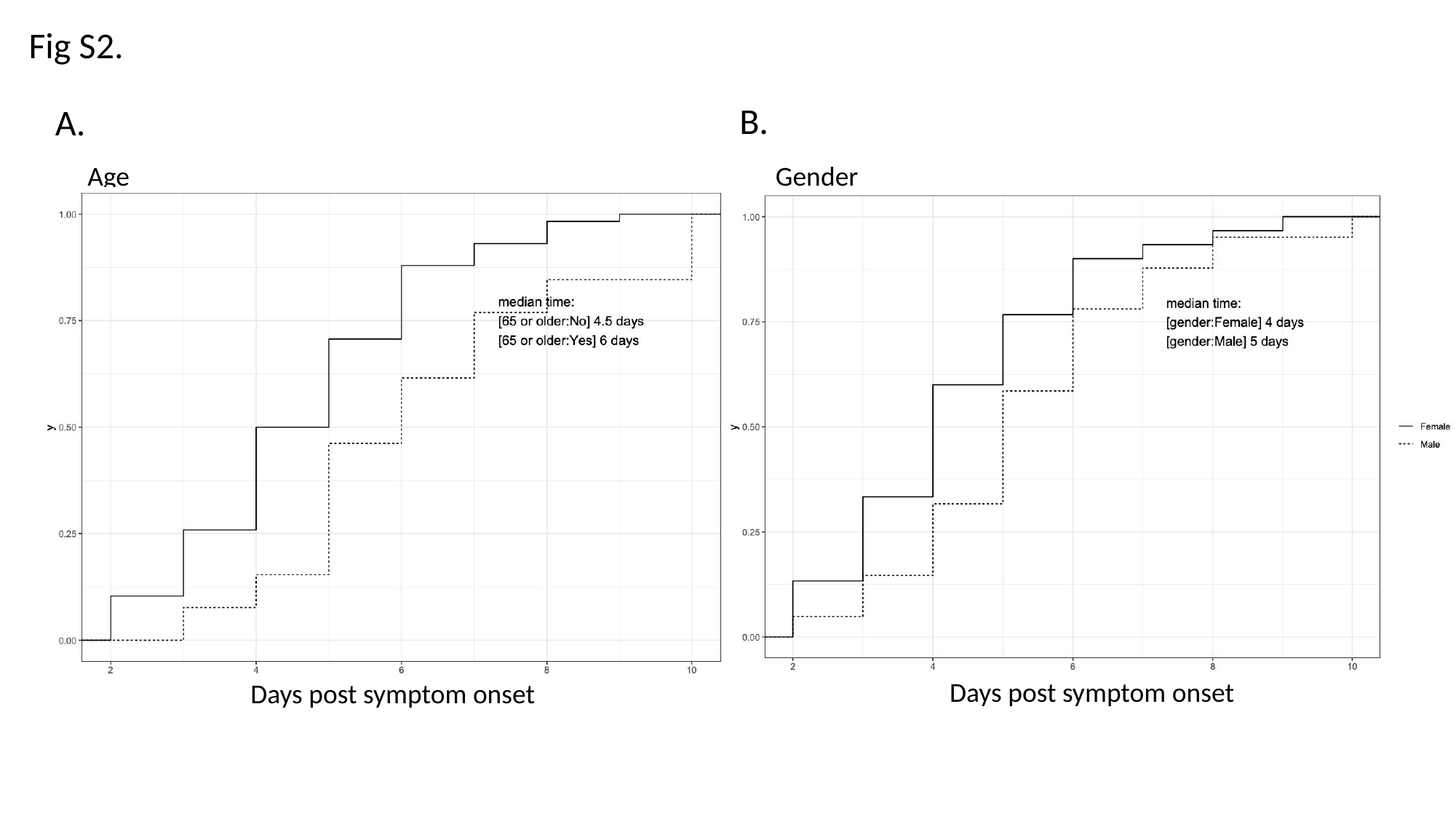

Fig S2.
B.
A.
Age
Gender
Days post symptom onset
Days post symptom onset

## Slide 3
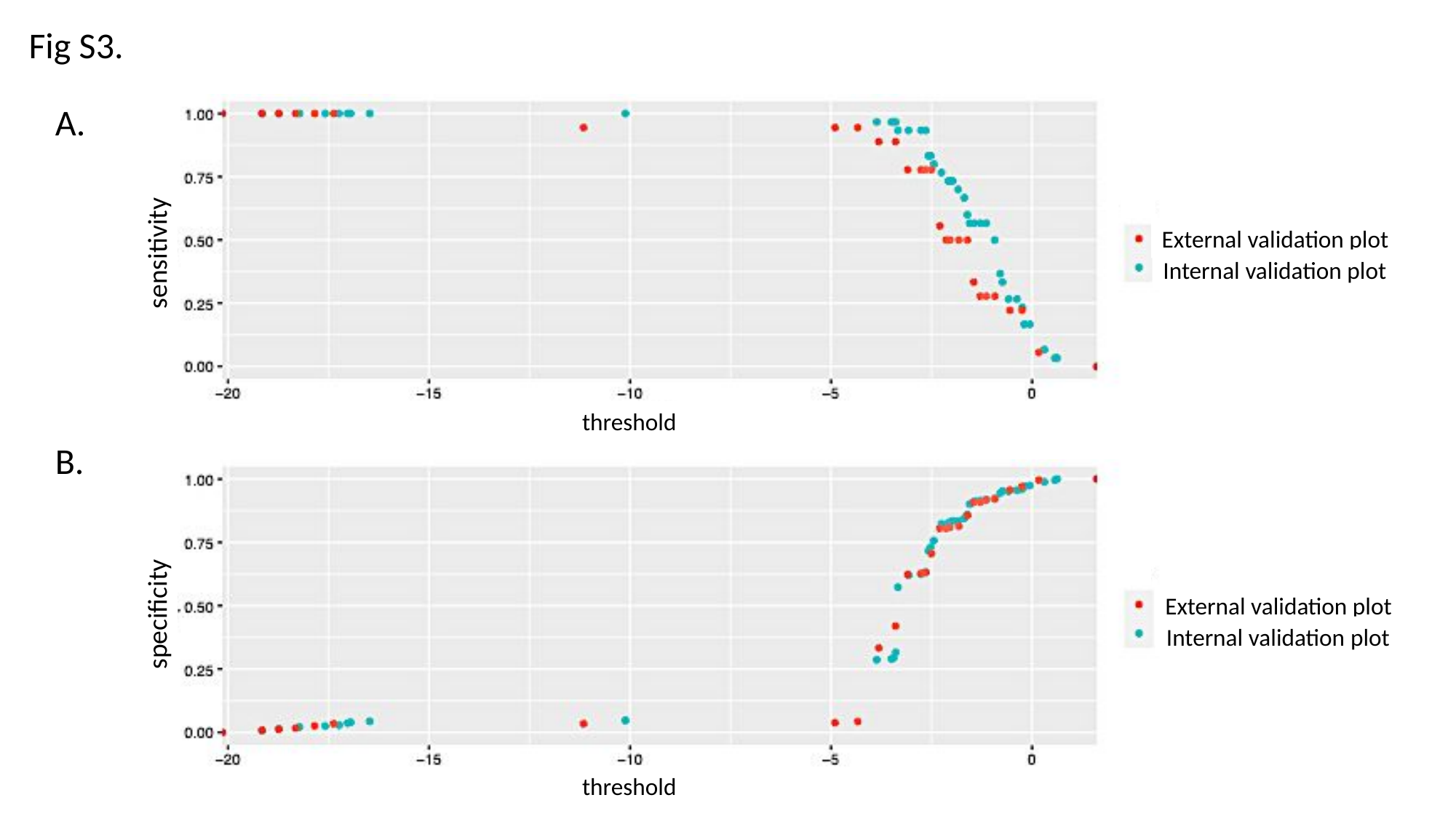

Fig S3.
A.
External validation plot
sensitivity
Internal validation plot
threshold
External validation plot
specificity
Internal validation plot
threshold
B.
